# Predicting hospitalisation-associated functional decline in older patients admitted to a cardiac care unit with cardiovascular disease: a prospective cohort study

**DOI:** 10.1186/s12877-020-01510-1

**Published:** 2020-03-20

**Authors:** Bastiaan Van Grootven, Anthony Jeuris, Maren Jonckers, Els Devriendt, Bernadette Dierckx de Casterlé, Christophe Dubois, Katleen Fagard, Marie-Christine Herregods, Miek Hornikx, Bart Meuris, Steffen Rex, Jos Tournoy, Koen Milisen, Johan Flamaing, Mieke Deschodt

**Affiliations:** 1 Research Foundation – Flanders (FWO), Brussels, Belgium; 2 Department of Public Health and Primary Care, KU Leuven - University of Leuven, Leuven, Belgium; 3 Department of Cardiovascular Medicine, University Hospitals Leuven, Leuven, Belgium; 4 Department of Geriatric Medicine, University Hospitals Leuven, Leuven, Belgium; 5 Department of Cardiovascular Sciences, KU Leuven - University of Leuven, Leuven, Belgium; 6 Department of Rehabilitation Sciences, KU Leuven - University of Leuven, University Hospitals Leuven, Leuven, Belgium; 7 Department of Anaesthesiology, University Hospitals Leuven, Leuven, Belgium; 8 Department of Public Health, Institute of Nursing Science, University of Basel, Basel, Switzerland

**Keywords:** Prognosis, Cohort, Aged, Prediction, Functional, Geriatric

## Abstract

**Background:**

Up to one in three of older patients who are hospitalised develop functional decline, which is associated with sustained disability, institutionalisation and death. This study developed and validated a clinical prediction model that identifies patients who are at risk for functional decline during hospitalisation. The predictive value of the model was compared against three models that were developed for patients admitted to a general medical ward.

**Methods:**

A prospective cohort study was performed on two cardiac care units between September 2016 and June 2017. Patients aged 75 years or older were recruited on admission if they were admitted for non-surgical treatment of an acute cardiovascular disease. Hospitalisation-associated functional decline was defined as any decrease on the Katz Index of Activities of Daily Living between hospital admission and discharge. Predictors were selected based on a review of the literature and a prediction score chart was developed based on a multivariate logistic regression model.

**Results:**

A total of 189 patients were recruited and 33% developed functional decline during hospitalisation. A score chart was developed with five predictors that were measured on hospital admission: mobility impairment = 9 points, cognitive impairment = 7 points, loss of appetite = 6 points, depressive symptoms = 5 points, use of physical restraints or having an indwelling urinary catheter = 5 points. The score chart of the developed model demonstrated good calibration and discriminated adequately (C-index = 0.75, 95% CI (0.68–0.83) and better between patients with and without functional decline (chi^2^ = 12.8, *p* = 0.005) than the three previously developed models (range of C-index = 0.65–0.68).

**Conclusion:**

Functional decline is a prevalent complication and can be adequately predicted on hospital admission. A score chart can be used in clinical practice to identify patients who could benefit from preventive interventions. Independent external validation is needed.

## Introduction

Up to one out of three older patients who are admitted to the hospital experience a new disability in their basic activities of daily living [1]. This hospitalisation-associated functional decline in bathing, dressing, walking, toileting, continence or eating is associated with prolonged hospitalisation, hospital readmission, nursing home admissions and decreased survival [1–3].

While some functional decline is caused by an acute illness, it is more often the result of an interaction between a patients’ vulnerability and hospitalisation factors [1]. Frail patients are more vulnerable to develop functional decline because of the immobility, forced dependency in self-care and polypharmacy associated with hospitalisation. The presence of geriatric syndromes, e.g., mobility or cognitive impairment, are important pre-illness determinants for the development of functional decline [1, 4–11]. Older patients admitted for acute cardiovascular disease are particularly vulnerable for functional decline because up to 60% of this group suffers from one or more geriatric syndromes on hospital admission [3].

Despite the high incidence of functional decline and the high prevalence of geriatric syndromes, acute cardiovascular care remains largely a diagnosis driven discipline, and often neglects the patients’ functional needs [12]. Clinical prediction models identify patients who are likely to experience functional decline and who may benefit from preventive rehabilitative interventions and follow-up. A review of the literature identified three clinical models that were developed in patients who were 70 years or older and who were admitted to a general medical ward [1, 9–11]. All three models assess the presence of geriatric syndromes and medical comorbidities on hospital admission using a scoring chart. These models discriminated poorly to adequately between patients with and without functional decline (Area Under the Curve of 0.65 to 0.78) [9–11]. The clinical usefulness of these models was not evaluated. To date, no clinical prediction model is available for patients who are admitted to a cardiac care unit with acute cardiovascular disease.

We therefore developed and validated a clinical model for the prediction of hospitalisation-associated functional decline in patients aged 75 years or older and who were admitted to a cardiac care unit with acute cardiovascular disease. The aim was to construct a risk score that is easily measured by healthcare professionals with no additional cost or resources than those available during routine care. As a secondary aim, we compared the performance of the developed model against the available models that were previously developed for patients admitted to a general medical ward.

## Methods

A prospective cohort study was performed on two cardiac care units of the University Hospitals Leuven, Belgium, between September 2016 and June 2017. Patients aged 75 years or older were recruited on admission if they were admitted for non-surgical treatment of an acute cardiovascular disease, had an expected length of stay of 3 days or longer, consented to participate and were able to complete the assessment. Patients who were admitted from another hospital or from the intensive care unit were excluded. The Medical Ethics Committee of the Leuven University Hospitals approved this study and written informed consent was obtained for all patients who consented to participate. The study adhered to the Declaration of Helsinki.

All data were assessed by three researchers using standardised assessment forms. Assessments on admission were performed within 72 h: 88% within 24 h and 99% within 48 h. Researchers were trained by performing paired bedside assessments and having case discussions until a 100% inter-rater agreement was observed. A meeting was organised every 3 months to confirm agreement.

### Baseline characteristics

Sample characteristics included age, gender, living situation (home, retirement home or nursing home) and medical diagnosis (heart failure, valvular heart disease, ischemic heart disease, arrhythmia or other).

### Outcome

Hospitalisation-associated functional decline was defined as the development of new or worse dependency in Activities of Daily Living (ADL) according to the Katz index when patients were discharged home. The Katz index was measured on admission to the unit and on the day that the patient was discharged home. Patients were asked whether they needed assistance for bathing, dressing, walking, continence, toileting, or feeding [13]. Assistance was scored on a three-point scale: independent, partially dependent, completely dependent. A change of one point on the scale was considered clinically relevant. Nurses and informal caregivers were asked to confirm the ADL status in patients with cognitive impairment.

### Development and validation of a prediction model

Predictors were a priori selected based on their association with functional decline in previous studies, [1, 4–11] and their clinical utility (i.e., easy to assess with no additional costs or resources than those available during routine care). The model complexity was a priori restricted to five predictors based on the assumption that we would observe a minimal of 50 events (assuming a conservative incidence of 27% based on the review of McCusker et al. [7]). This allows for a more precise estimate of the coefficients for the risk score and prevents against overfitting of the model.

The predictors were assessed within the first 72 h of hospital admission. Mobility impairment was defined as the use of a walking aid before hospital admission as reported by the patient. Cognitive impairment was defined as a Mini-Cog score < 3 out of 5 points. The Mini-Cog assesses performance on two cognitive tasks, i.e., three-item word recall and clock drawing test. A higher score indicates a better cognitive performance [14]. The presence of depressive symptoms was defined as a score > 3 on the 10-item version of the geriatric Depression Scale. Patients were asked whether they experienced any of the 10 symptoms in the past week. Sum scores vary between 0 and 10 with higher scores indicating more depressive symptoms [15]. Loss of appetite was defined as self-reported loss of appetite in the past 3 months and was used as a proxy for risk for malnutrition. Use of restraints was defined as the use of physical restraints (e.g., vests, limb ties or chairs with restraints) or an indwelling urinary catheter between admission to the unit and assessment of the predictors [16]. Bed rails were not considered a restraint.

A multivariate logistic regression model was built using a full model approach. Unadjusted and adjusted Odds Ratios (OR) and regression coefficients were calculated with 95% Confidence Intervals (CI). Coefficients were shrinked to compensate for potential overfitting of the model. A uniform shrinkage factor was calculated based on the Chi^2^ and the degrees of freedom (df) of the model *((model Chi*^*2*^*– df)/model Chi*^*2*^*)* [17]. Multicollinearity was evaluated using the Variance Inflation Factor and Tolerance values. A score chart for the prediction model was developed by multiplying the shrinked coefficients with 10 and rounding the score [18].

Discrimination was assessed using the C-index. We performed an internal validation of the model using nonparametric bootstrapping samples (*n* = 1000). This procedure estimates the optimism of developing and validating the prediction model in the same sample of patients, and provides a bias-corrected measure for model discrimination.

### Comparison with existing prediction models

Three clinical prediction models for hospitalisation-associated functional decline were previously developed in patients admitted to a general medical ward (see Table S1 and S2 in the supplementary material). Each model was scored within the first 72 h of hospital admission (see Table S3 in the supplementary material for definitions of all predictors). The model by Inouye et al. defines the presence of a decubitus ulcer, cognitive impairment, functional impairment and low social activity level as predictors [9]. The model by Mehta et al. defines age, premorbid dependency on instrumental activities of daily living and basic ADL, inability to run a short distance, inability to walk stairs, metastatic cancer or stroke, cognitive impairment and albumin level as predictors [10]. The model by Sager et al. defines age, cognitive impairment and premorbid dependency on instrumental ADL as predictors [11].

The discrimination of the models was compared using the chi^2^ test for equality for two or more Receiver Operating Characteristic areas. Calibration was assessed using the Hosmer-Lemeshow Goodness of Fit test and by constructing calibration plots. The clinical usefulness was assessed using classification statistics (sensitivity, specificity, predicted values). For all models, the cut-off value, that identifies if a patient is considered at risk, was based on the Youden index to allow for equal comparison between the models [19]. The Youden index corresponds to the cut-off value with the optimal combination of sensitivity and specificity.

### Missing data

There were 53 cases (28%) with missing data for the Mehta et al. model because of missing albumin levels, which were not routinely assessed on hospital admission. There was no significant relationship between missing data and hospitalisation-associated functional decline (OR 1.8, 95% CI (0.9–3.5)). We therefore assumed that data were missing completely at random [20]. Multiple imputation (M = 5) with all predictors, the outcome and auxiliary variables was performed to allow comparison between models with an equal number of subjects. A parametric multiple linear regression model was used as the data were normally distributed. A sensitivity analysis with a complete case analysis was performed to evaluate the influence of data imputation.

### Post-hoc analysis

After the model was developed and validated, we used a nested model approach to investigate if adding the predictor ‘age’ improved the discrimination. We also performed a chi-squared test to compare the C-index estimates per age group (75 to 80, 81 to 84, or > 84).

## Results

### Sample characteristics

A total of 930 patients were screened for inclusion, 244 patients were eligible and 189 patients consented to participate. The mean age was 84 years and the largest group of patients (37%) had acute decompensated heart failure (see Table 1). Half of the patients had a mobility impairment and 40% reported a loss of appetite before hospital admission, one in three patients had a cognitive impairment and one in four patients reported depressive symptoms or was restrained. In total, 33% of the patients developed functional decline during hospitalisation.

**Table 1:** Sample characteristics

Sample characteristics	Sample (***n*** = 189)
Age, mean (SD)	84 (5)
Male gender, n (%)	100 (53)
Medical diagnosis, n (%)	
Heart failure	70 (37)
Valvular heart disease	8 (4)
Ischemic heart disease	30 (16)
Arrhythmia	46 (24)
Other	35 (19)
Living situation, n (%)	
At home	170 (90)
Retirement home	6 (3)
Nursing home	13 (7)
Katz index of ADL, mean (SD)	
On admission	8.6 (2.7)
On discharge	8.8 (2.8)
Functional decline, n (%)	63 (33)
Length of stay (days), mean (SD)	9 (6)
Predictors for functional decline, n (%)	
Mobility impairment: use of ambulatory device	106 (56)
Cognitive impairment: Mini-Cog score < 3/5	62 (33)
Depressive symptoms: GDS score > 3/10	47 (25)
Loss of appetite in past 3 months	75 (40)
Use of physical restraints or indwelling urinary catheter	46 (24)

Abbreviations: *SD* Standard Deviations, *ADL* Activities of Daily Living, *GDS* Geriatric Depression Scale;

### Performance and validation of the prediction model

All predictors increased the odds for functional decline but only mobility impairment, cognitive impairment and loss of appetite were statistically significant (see Table 2).

**Table 2:** Developing of a prediction score for hospitalisation-associated functional decline

Predictors	Estimation of main effects		Prediction model ^a^		
	*Unadjusted OR (95% CI)*	*Adjusted OR (95% CI)*	*Coefficients (95% CI)*	*Shrinked coefficients*^*b*^	*Score chart*^*c*^
Mobility impairment	3.75 (1.91–7.39)	2.81 (1.37–5.77)	1.03 (0.31–1.75)	0.88 (0.26–1.49)	9
Cognitive impairment	2.97 (1.57–5.62)	2.32 (1.16–4.62)	0.84 (0.15–1.53)	0.71 (0.13–1.30)	7
Loss of appetite	2.69 (1.44–5.01)	2.14 (1.08–4.22)	0.76 (0.08–1.44)	0.64 (0.07–1.22)	6
Depressive symptoms	2.17 (1.10–4.27)	1.70 (0.80–3.59)	0.53 (− 0.22–1.28)	0.45 (−0.19–1.09)	5
Use of restraints	2.58 (1.30–5.10)	1.76 (0.84–3.70)	0.57 (− 0.18–1.31)	0.49 (−0.15–1.11)	5

Abbeviations: *OR* Odds Ratio, *CI* Confidence Interval;

^a^ Model evaluation: Discrimination: C-index = 0.76, 95% CI (0.68–0.83); Discrimination after internal validation: C-index = 0.73, 95% CI (0.65–0.80); Calibration: Hosmer-Lemeshow goodness of fit Chi^2^ = 8.07, *p* = 0.362; Assumptions: mean VIF = 1.09, VIF < 10 and tolerance > 0.1 was observed for all variables;

^b^ Coefficients were shrinked using a uniform shrinkage factor ((34.32–5)/ 34.32) = 0.85;

^c^ Score chart was developed by multiplying the shrinked coefficients with 10 and rounding the score;

The full model with five predictors discriminated adequately between patients with and without functional decline (C-index = 0.76, 95% CI (0.68 to 0.83)) and was well calibrated (*p* = 0.326; i.e., patients considered to have a low risk had a low probability for functional decline and patients with a high risk had a high probability for functional decline). After internal validation, the discrimination remained adequate (C-index = 0.73, 95% CI (0.65–0.80)).

A score chart was developed by estimating points for each predictor based on their regression coefficients (see Table 2). The discrimination remained adequate (C-index = 0.75, 95% CI (0.68–0.83) with good calibration (*p* = 0.499).

In the nested model, age was not a significant predictor for functional decline (OR = 1.00, 95% CI (0.93–1.08), and it did not improve the ‘fit of the model’ (*p* = 0.958). The discrimination remained the same (C-index = 0.75, 95% CI (0.68–0.82)). The discrimination was statistically not different between age groups (*p* = 0.395).

### Comparison of prediction models

All models increased the odds for developing functional decline during hospitalisation (see Table 3). The previously developed models discriminated poorly (range C-index of models = 0.65–0.68) between patients with and without functional decline. After internal validation, the discrimination of the new model remained better than that of the previous models when comparing the C-Index (chi^2^ = 12.8, *p* = 0.005). Based on the calibration plots (see Fig. S1 in the supplementary material), the calibration was adequate for the new model and the model of Inouye et al., and was poor for the models of Mehta et al. and Sager et al.

**Table 3:** Evaluation of clinical models for predicting hospitalisation-associated functional decline

Model evaluation	Prediction models			
	*Developed model*	*Inouye* et al. *1993*	*Mehta,* et al. *2011*^*b*^	*Sager* et al. *1996*
OR (95% CI)	1.12 (1.08–1.17)	2.44 (1.56–3.81)	1.26 (1.12–1.41)	1.62 (1.24–2.12)
Discrimination ^a^	0.75 (0.68–0.83)	0.67 (0.59–0.74)	0.68 (0.61–0.76)	0.65 (0.57–0.73)
Calibration	Chi^2^ = 6.35, *p* = 0.499 ^c^	Chi^2^ = 0.11, *p* = 0.948 ^d^	Chi^2^ = 11.80, *p* = 0.0667 ^e^	Chi^2^ = 11.80, *p* = 0.0667 ^f^
Clinical usefulness	Cutoff value = 13 Sensitivity = 71% Specificity = 70% PPV = 54% NPV = 83% Correctly classified = 70%	Cutoff value = 1 Sensitivity = 46.0% Specificity = 80.2% PPV = 54% NPV = 75% Correctly classified = 69%	Cutoff value = 4 Sensitivity = 65% Specificity = 60% PPV = 45% NPV = 78% Correctly classified = 62%	Cutoff value = 4 Sensitivity = 60% Specificity = 61% PPV = 44% NPV = 76% Correctly classified = 61%

Abbreviations: *PPV* Positive Predictive value, *NPV* Negative Predictive Value, *CI* Confidence Interval;

^a^ Discrimination was assessed using the C-index, and models were compared using the chi^2^ test for equality for two or more Receiver Operating Characteristic areas: chi^2^ = 12.8, *p* = 0.005;

^b^ Sensitivity analysis for Mehta et al. 2011 using a complete case analysis instead of multiple imputation: OR = 1.18 (1.03–1.36); Discrimination: 0.63, 95% CI (0.54–0.73); Calibration: chi^2^ = 4.27, *p* = 0.640;

Calibration was assessed using the Hosmer – Lemeshow goodness of fit test. The goodness of fit could only be assessed in quantiles of ^c^ 9 groups, ^d^ 4 groups, ^e^ 8 groups and ^f^ 5 groups because of ties in the data;

The clinical usefulness was comparable for all models with high negative predictive values ranging from 83 to 76% and low positive predictive values ranging from 54 to 44%. The overall classification was best for the new model (70%) and the model by Inouye et al. (69%) compared against the models by Mehta et al. (62%) and Sager et al. (61%).

## Discussion

Because no prediction model for hospitalisation-associated functional decline was available for older cardiac patients, this study developed a new prediction model and compared it against three models that were previously developed for patients who were admitted to a general medical ward.

The majority of patients in our cohort had at least one geriatric syndrome on hospital admission (see Table 1) and one in three patients developed functional decline during hospitalisation. The newly developed model uses mobility and cognitive impairment, depressive symptoms, the loss of appetite and the use of physical restraints or indwelling urinary catheters as predictors for functional decline. In the four prediction models, mobility or functional and cognitive impairment were consistent predictors for hospitalization-associated functional decline. Mobility impairment was the strongest predictor. All models confirm a dose response relationship: the more predictors that were present, the higher the risk score, and the higher the probability for functional decline (see Table S4 in the supplementary material). This dose response relationship between the presence of geriatric syndromes and the development of functional dependency in community dwelling older adults [21], and for the onset of delirium in hospitalized older adults was previously observed [22].

After internal validation, the new model discriminated better than the previous models but the calibration plots indicate that the risk for functional decline was consistently underestimated in all models. This may indicate that an important predictor may be missing. For example, medical comorbidities and illness severity have also demonstrated an association with functional decline [23]. These variables were not considered in the model because they require diagnostic interviewing and testing and may be too complex for clinical practice. Furthermore, we only considered a static prediction on hospital admission but the probability for functional decline changes during hospitalisation. For example, the onset of delirium has been associated with functional decline [24]. Dynamic predictions can adjust the probability for functional decline using repeated assessments, but this was not investigated.

Predicting functional decline on hospital admission can offer several advantages. First, resources can be used more efficiently and effectively because high-risk patients have a higher absolute benefit from interventions to prevent functional decline than low-risk patients do. Second, it facilitates a proactive approach and shifts the focus to the prevention of functional decline and its negative consequences for the older patient. A meta-analysis of randomized controlled trials studying the effects inpatient rehabilitation observed improved functional status in geriatric patients [25]. Programs were more effective when a comprehensive geriatric assessment (CGA), defined as a “multidimensional, interdisciplinary diagnostic process to determine the medical, psychological and functional capabilities of an older person with frailty, followed by the implementation of a coordinated and integrated plan for treatment and follow-up” was used to tailor the rehabilitation to the patients’ needs [26]. The four prediction models all assess basic aspects of a CGA and can in this way be used to select patients in need for follow-up. To the best of our knowledge, a CGA-based program has not yet been evaluated in older patients on a cardiac care unit.

All current models result in substantial number of false positive predictions. For the purpose of this paper, the Youden index was used to illustrate the clinical usefulness. However, the Youden index assumes that false positive and false negative predictions are equally good or bad. For the use in practice, the ‘at-risk cut-off score’ should be decided on the individual context. When there are sufficient resources for the follow-up of at risk patients, a lower cut-off score can be used at the expense of more false positive predictions. A follow-up assessment can further select patients that could benefit from rehabilitation. When resources are scarce, a higher cut-off score will minimise false positive predictions. Clinicians interested in using a prediction model can find the probabilities, sensitivity and specificity for functional decline for each cut-off score of each model in Table S4 in the supplementary materials.

The prospective data collection using standardised assessments by trained researchers and a comprehensive evaluation of the four models contributed to the strengths of this study. However, some considerations should be noted. First, an independent data set was not available to validate the newly developed model. As a result, the performance of the developed model may be too optimistic. This may have biased the comparison of the discrimination between the models in favor of the developed model. However, several measures were used to minimise this bias: 1) predictors were selected based on a review of the literature and not based on *p*-values. A stepwise selection of predictors using *p*-values overestimates the regression coefficients and model performance [27]; 2) the number of predictors were a priori restricted to five and coefficients were shrunk to minimise overfitting of the model; and 3) bootstrapping methods were used to adjust the discrimination for bias. Second, there is considerable heterogeneity in how predictor variables are defined and assessed in practice. Using a different instrument may decrease (or increase) the performance of a prediction model. We tried to decrease the dependency of predictions for a specific scale by dichotomising predictors using validated cut-off values. For example, the presence of cognitive and functional impairment are key predictors in the different models even when different instruments are used. However, dichotomising the predictors may also have resulted in less accurate estimates of predictor scores leading to a less accurate discrimination and calibration. Third, selection of predictors is a balance between methodological and clinical considerations and therefore to a certain extent subjective. Nonetheless, the developed model demonstrates adequate discrimination, which confirms that we selected appropriate predictors.

Future research should focus on an independent external validation of the developed model, expanding the model with predictors that can be easily assessed in clinical practice and interventions that effectively prevent functional decline in older patients admitted to a cardiac care unit with acute cardiovascular disease. Interesting areas for exploration are the use of dynamic predictions and the comparison of the prediction model with a frailty assessment. The use of electronic health records to identify frailty profiles may be particularly promising to identify patients at risk for functional decline [28]. However, validation in the acute care setting is currently lacking.

## Conclusion

Geriatric syndromes are prevalent on hospital admission in patients who are hospitalised on a cardiac care unit with cardiovascular disease and can adequately predict hospitalisation-associated functional decline. Cognitive and mobility impairment are key predictors for decline. A prediction model appears to have clinical value for selecting patients who could benefit from rehabilitation, but false positive predictions should be considered. Independent external validation is needed.

## Supplementary information

**Additional file 1: Table S1.** Characteristics of studies identified in the literature. **Table S2.** Performance of studies identified in the literature. **Table S3.** Assessment of predictors in the cohort study of models that were identified in the literature. **Figure S1.** Calibration plots for predicting hospitalisation-associated functional decline. **Table S4.** Probability for hospitalisation-associated functional decline with corresponding sensitivity and specificity for observed cut-off values of prediction models.

## Data Availability

The datasets used and/or analysed during the current study are available from the corresponding author on reasonable request.

## Abbreviations

ADL: Activities of Daily Living
OR: Odds Ratios
df: Degrees of Freedom
CGA: Comprehensive Geriatric Assessment
