# Supplementary material for: Predicting hospitalisation-associated functional decline in older patients admitted to a cardiac care unit with cardiovascular disease: a prospective cohort study
Source: BMC Geriatr. 2020 Mar 20;20:112. doi: 10.1186/s12877-020-01510-1 (PMC7082946; doi:10.1186/s12877-020-01510-1)
Supplement: Supplementary file 1 — Additional file 1: Table S1. Characteristics of studies identified in the literature. Table S2. Performance of studies identified in the literature. Table S3. Assessment of predictors in the cohort study of models that were identified in the literature. Figure S1. Calibration plots for predicting hospitalisation-associated functional decline. Table S4. Probability for hospitalisation-associated functional decline with corresponding sensitivity and specificity for observed cut-off values of prediction models. [file 12877_2020_1510_MOESM1_ESM.docx]

**Supplementary files**

Table S1. Characteristics of studies identified in the literature

Table S2. Performance of studies identified in the literature

Table S3. Assessment of predictors in the cohort study of models that were identified in the literature

Figure S1. Calibration plots for predicting hospitalisation-associated functional decline

Table S4. Probability for hospitalisation-associated functional decline with corresponding sensitivity and specificity for observed cut-off values of prediction models

**Table S1 Characteristics of studies identified in the literature**

| **Study characteristics** | **Clinical prediction models identified in the literature** | | |
| --- | --- | --- | --- |
|  | *Inouye et al. 1993* | *Mehta, et al. 2011* | *Sager et al. 1996* |
| Study design | Two prospective cohort studies: development and validation | Prospective cohort study and secondary analysis of randomised controlled trial | Secondary analysis from prospective cohort study and clinical trial |
| Setting | Teaching hospital, Connecticut (USA) | Two teaching hospitals, Ohio (USA) | Six hospitals: medical centers and university hospitals in California, Ohio, Wisconsin, Illinois, Connecticut (USA) |
| Study dates | Development: Nov 1989 to June 1990  Validation: July 1990 to July 1991 | 1993 to 1998 | Development: 1990 to 1992  Validation: 1989 to 1990 |
| Sample size | Development: 188  Validation: 142 | Development: 885  Validation: 753 | Development: 597  Validation: 783 |
| Participants | Patients aged 70 years or older and admitted to medical service  - Age development cohort: 78.4 (5.8)  - Age validation cohort: 78.1 (6.0) | Functionally independent patients aged 70 years or older and admitted to medical services  - Age development cohort: 78 (6)  - Age validation cohort: 79 (7) | Noninstitutionalised patients aged 70 years or older and admitted to medical services and who survived three months after discharge  - Age development cohort: 79 (6.2)  - Age validation cohort: 80 (6.1) |
| Outcome | Nurses recorded new decline occurring in the hospital and persisting until discharge in five Activities of Daily Living (feeding, bathing, grooming, dressing, toileting) | Self-reported new dependency in one or more Activities of Daily Living of the Katz Index at hospital discharge (bathing, dressing, transferring, toileting, eating) | Self-reported new dependency in one or more Activities of Daily Living at hospital discharge (bathing, dressing, transferring, walking, toileting, eating) |
| Blinding | Assessors blinded to research question | Not reported | Not reported |
| Selection of predictors | 1) A-priori clinical relevance  2) Relative Risk ≥ 1.5  3) Statistical significance at p < 0.05 | 1) Studies or risk factors  2) Conceptual models of disablement | Not reported |
| Missing data | 5 cases with missing data  Complete case analysis | For most variables, missing < 2%  Multiple imputation | 135 (23%) cases in development cohort  185 (33%) cases in validation cohort  Complete case analysis |
| Statistical analysis | Proportional Hazard model: forward stepwise procedure: p < 0.1 enter, p > 0.15 remove | Logistic regression: best subset regression procedure creating range of models with 1 to 20 predictors. Final selection based on clinical relevance | Logistic regression: selection based on p < 0.05 |
| Score chart | One point per predictor | Points for each predictor assigned by dividing regression coefficient with lowest coefficient in model and rounding to nearest integer | Scoring system based on parameter estimates of regression model and their relative contribution to loss of ADL function |

**Table S2. Performance of studies identified in the literature**

| **Model performance** | **Clinical prediction models** | | |
| --- | --- | --- | --- |
|  | *Inouye et al. 1993* | *Mehta, et al. 2011* | *Sager et al. 1996* |
| Score chart | Decubitus ulcer = 1  Cognitive impairment = 1  Functional impairment = 1  Low social activity level = 1 | Age  - Age 80 – 89 = 1  - Age > 89 = 2  Dependent on > 2 IADLs = 2  Mobility  - Unable to run short distance = 1  - Unable to walk stairs or uphill = 2  ADL dependencies  - 2 – 3 ADL dependencies = 1  - 4 – 5 ADL dependencies = 3  Metastatic cancer/stroke = 2  Severe cognitive impairment = 1  Albumin < 3.0 g/dL = 2 | Age  - Age 75 – 84 = 1  - Age > 84 = 2  Cognitive impairment = 1  IADL score 0 – 5 = 2 |
| Discrimination | Not reported | Development = 0.78  Validation = 0.78 | Development = not reported  Validation = 0.65 |
| Calibration | Not reported | Development p = 0.4  Validation p = 0.54 | Not reported |
| Clinical usefulness | Not reported | Not reported | Not reported |

Abbreviations: IADL = Instrumental Activities of Daily Living; ADL = Activities of Daily Living

Discrimination was assessed using the C-index or Area Under the Curve; Calibration was assessed using the Hosmer – Lemeshow goodness of fit test;

**Table S3. Assessment of predictors in the cohort study of models that were identified in the systematic review**

| **Predictors** | **Study** | **Definition of assessment in original paper** | **Definition of assessment in current validation study** |
| --- | --- | --- | --- |
| Decubitus ulcer | Inouye et al 1993 | Standardised skin check for breakdown at 11 pressure points | Nurses recorded presence of decubitus ulcer in electronic patient record |
| Cognitive impairment | Inouye et al 1993 | Mini-Mental State Examination < 20 | Mini-Cog < 3 |
|  | Mehta et al. 2011 | Five or more errors on the Short Portable Mental Status Questionnaire |  |
| Functional impairment | Inouye et al 1993 | Self-reported (in)dependency with at least one of seven ADLs two weeks before admission: feeding, bathing, grooming, dressing, toileting, transferring, walking | Self-reported (in)dependency with at least one of seven ADLs two weeks before admission: feeding, bathing, grooming, dressing, toileting, transferring, walking |
|  | Mehta et al. 2011 | Self-reported (in)dependency with 1) two or three and 2) four or five of five ADLs on hospital admission: bathing, dressing, transferring, toileting, eating | Self-reported (in)dependency with 1) two or three and 2) four or five of five ADLs on hospital admission: bathing, dressing, transferring, toileting, eating |
|  | Mehta et al. 2011 | Self-reported (in)dependency in three or more of seven IADLs two weeks prior to hospital admission: telephone, transportation, shopping, preparing meals, doing housework, taking medicines, handling money | Self-reported (in)dependency in three or more of seven IADLs two weeks prior to hospital admission: telephone, transportation, shopping, preparing meals, doing housework, taking medicines, handling money |
| Mobility impairment | Mehta et al. 2011 | Self-reported ability to walk uphill or stairs | Self-reported ability to walk stairs |
|  | Mehta et al. 2011 | Unable to run a short distance | Self-reported ability to walk outside the house |
| Low social activity level | Inouye et al 1993 | Participation in three or fewer of 11 representative social activities in a typical month, including religious and group activities (e.g. senior center, community group), outings (e.g. movies, plays, restaurants, sporting events), hobbies, sports, bingo or games and paid or volunteer work | Patient self-reported social isolation |
| Age | Mehta et al. 2011 | Patients aged 80 – 89  Patients aged > 89 | Patients aged 80 – 89  Patients aged > 89 |
| Metastatic cancer or stroke | Mehta et al. 2011 | Clinical diagnosis | Clinical diagnosis in medical record |
| Albumin < 3g/dL | Mehta et al. 2011 | Laboratory analysis | Laboratory analysis in medical record |

Abbreviations: IADL = Instrumental Activities of Daily Living; ADL = Activities of Daily Living

**Figure S1 Calibration plots for predicting hospitalisation-associated functional decline**

**
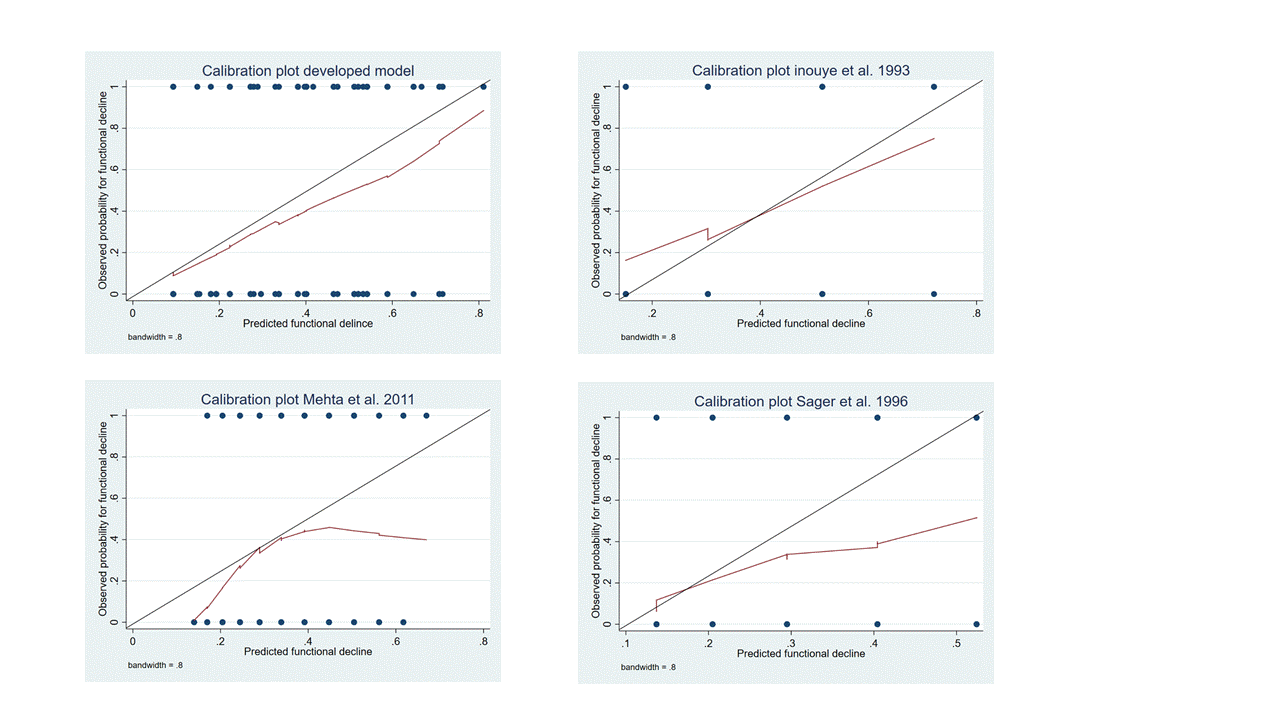
**

**Table S4. Probability for hospitalisation-associated for observed cut-off values of prediction models**

| **Cutoff score** | **Developed model** | | | **Inouye et al. 1993** | | | **Mehta, et al. 2011** | | | **Sager et al. 1996** | | |
| --- | --- | --- | --- | --- | --- | --- | --- | --- | --- | --- | --- | --- |
|  | *Prob* | *Sens* | *Spec* | *Prob* | *Sens* | *Spec* | *Prob* | *Sens* | *Spec* | *Prob* | *Sens* | *Spec* |
| 0 | 0.09 | 1.0 | 0.0 | 0.0 | 1.0 | 0.0 | 0.14 | 1.0 | 0.08 |  |  |  |
| 1 | - | - | - | 0.15 | 1.0 | 0.0 | 0.17 | 0.97 | 0.25 | 0.14 | 0.97 | 0.24 |
| 2 | - | - | - | 0.51 | 0.46 | 0.80 | 0.20 | 0.94 | 0.36 | 0.20 | 0.87 | 0.29 |
| 3 | - | - | - | 0.72 | 0.04 | 0.99 | 0.24 | 0.84 | 0.50 | 0.29 | 0.60 | 0.61 |
| 4 | - | - | - | - | - | - | 0.29 | 0.65 | 0.60 | 0.40 | 0.27 | 0.87 |
| 5 | 0.15 | 0.92 | 0.33 | - | - | - | 0.34 | 0.54 | 0.69 | 0.52 | 0.0 | 1.0 |
| 6 | 0.18 | 0.90 | 0.37 | - | - | - | 0.39 | 0.41 | 0.75 | - | - | - |
| 7 | 0.19 | 0.90 | 0.43 | - | - | - | 0.45 | 0.22 | 0.85 | - | - | - |
| 8 | - | - | - | - | - | - | 0.50 | 0.11 | 0.91 | - | - | - |
| 9 | 0.22 | 0.83 | 0.56 | - | - | - | 0.56 | 0.06 | 0.96 | - | - | - |
| 10 | - | - | - | - | - | - | 0.62 | 0.02 | 1.0 | - | - | - |
| 11 | 0.27 | 0.78 | 0.60 | - | - | - | 0.67 | 0.0 | 1.0 | - | - | - |
| 12 | 0.30 | 0.75 | 0.62 | - | - | - | - | - | - | - | - | - |
| 13 | 0.34 | 0.71 | 0.70 | - | - | - | - | - | - | - | - | - |
| 14 | 0.34 | 0.71 | 0.67 | - | - | - | - | - | - | - | - | - |
| 15 | 0.38 | 0.60 | 0.77 | - | - | - | - | - | - | - | - | - |
| 16 | 0.40 | 0.51 | 0.79 | - | - | - | - | - | - | - | - | - |
| 17 | 0.42 | 0.49 | 0.79 | - | - | - | - | - | - | - | - | - |
| 18 | 0.46 | 0.46 | 0.82 | - | - | - | - | - | - | - | - | - |
| 19 | 0.46 | 0.48 | 0.80 | - | - | - | - | - | - | - | - | - |
| 20 | 0.52 | 0.40 | 0.88 | - | - | - | - | - | - | - | - | - |
| 21 | 0.54 | 0.29 | 0.94 | - | - | - | - | - | - | - | - | - |
| 22 | 0.59 | 0.21 | 0.96 | - | - | - | - | - | - | - | - | - |
| 23 | - | - | - | - | - | - | - | - | - | - | - | - |
| 24 | - | - | - | - | - | - | - | - | - | - | - | - |
| 25 | 0.65 | 0.16 | 0.97 | - | - | - | - | - | - | - | - | - |
| 26 | 0.67 | 0.14 | 0.97 | - | - | - | - | - | - | - | - | - |
| 27 | 0.71 | 0.10 | 0.99 | - | - | - | - | - | - | - | - | - |
| 28 | - | - | - | - | - | - | - | - | - | - | - | - |
| 29 | - | - | - | - | - | - | - | - | - | - | - | - |
| 30 | - | - | - | - | - | - | - | - | - | - | - | - |
| 31 | - | - | - | - | - | - | - | - | - | - | - | - |
| 32 | 0.81 | 0.0 | 1.0 | - | - | - | - | - | - | - | - | - |

Abbreviations: Prob = Probability; Sens = Sensitivity; Spec = Specificity;
